# More dreams of the rarebit fiend: food sensitivity and dietary correlates of sleep and dreaming

**DOI:** 10.3389/fpsyg.2025.1544475

**Published:** 2025-07-01

**Authors:** Tore Nielsen, Jade Radke, Claudia Picard-Deland, Russell Arnold Powell

**Affiliations:** ^1^Dream & Nightmare Lab, Center for Advanced Research in Sleep Medicine, CIUSSS-NIM, Montréal, QC, Canada; ^2^Département de Psychiatrie et Addictologie, Université de Montréal, Montréal, QC, Canada; ^3^Department of Psychology, University of British Columbia, Vancouver, BC, Canada; ^4^Department of Psychology, Grant MacEwan University, Edmonton, AB, Canada

**Keywords:** dreaming, nightmares, sleep quality, diet, lactose intolerance, food allergy, night-eating

## Abstract

**Background:**

Despite centuries-old beliefs and anecdotal evidence that food can influence one's sleep and dreams—an example being the classic *Dream of the Rarebit Fien*d cartoon series—the topic has only rarely been researched.

**Methods:**

We asked 1,082 participants to complete an online survey to test specific hypotheses on why people perceive that food affects their dreams, including whether specific foods influence dreams directly (food-specific effects), through physiological symptoms (food distress), or via altered sleep quality (sleep effects). Survey measures included standard demographic variables, targeted probes about self-perceived effects of specific foods on dreams, questions about diet, food intolerances and allergies, personality questionnaires, measures of sleep quality (Pittsburgh Sleep Quality Index) and the Nightmare Disorder Index.

**Results:**

A total of 40.2% of participants reported that certain foods either worsened (24.7%) or improved (20.1%) their sleep, while 5.5% of participants reported that food affected their dreams. The perceived effect of food on dreams was associated with higher nightmare recall and Nightmare Disorder Index scores, with changes being blamed primarily on desserts/sweets (31%) and dairy (22%). The effect was also associated with food allergies and Gluten Intolerance, while worse sleep perceptions were tied to Lactose Intolerance. Nightmare Disorder Index scores were strongly associated with Food Allergy and Lactose Intolerance, the latter being mediated by the severity of gastrointestinal symptoms. Healthy eating, such as less evening eating, predicted higher dream recall, while unhealthy eating—including gastric symptoms, lower reliance on hunger and satiety cues, and evening eating—predicted nightmares and dream negativity.

**Conclusions:**

These results support the food-specific effects, food distress, and sleep effects hypotheses to varying degrees. They replicate associations between diet and dream features, highlighting food sensitivities, particularly Lactose Intolerance, as contributors to nightmare prevalence. Findings open new avenues of research on food-dependent dreaming by suggesting dairy-induced gastrointestinal symptoms as one plausible basis for bizarre or disturbing dreams. They have clear implications for understanding how dietary factors may influence sleep quality and the occurrence of nightmares and could inform non-pharmacological interventions for sleep disturbances.

## 1 Introduction

**Figure 1:**
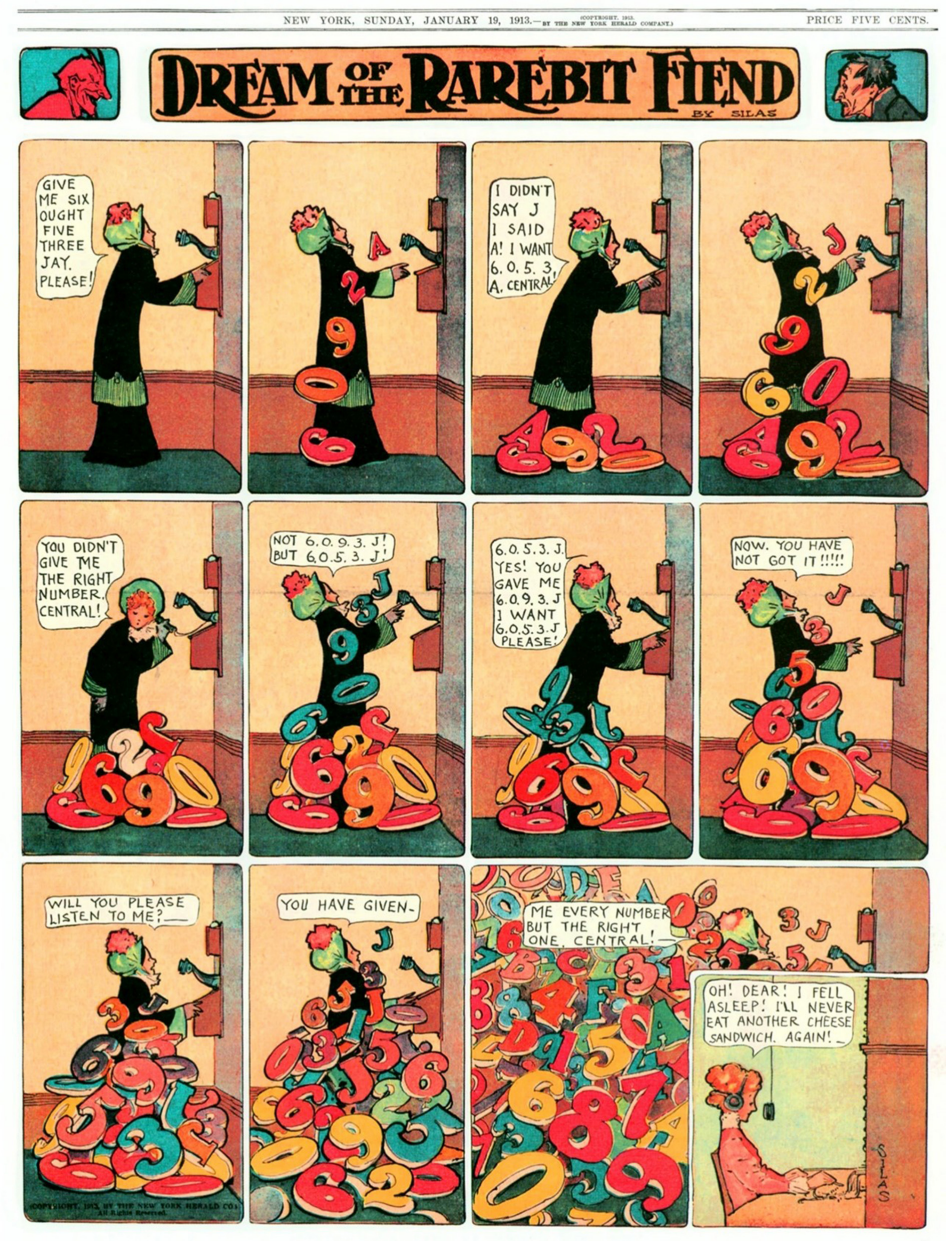
Protagonists in the cartoons of Winsor McCay frequently blamed their presleep ingestion of cheese dishes for occurrences of bizarre dream and nightmare events. (Reproduced from “Dream of the Rarebit Fiend” by Windsor McCay, Dream of the Rarebit Fiend by Winsor McCay : Winsor McCay : Free Download, Borrow, and Streaming : Internet Archive, CC0).

People have long believed in the power of food to influence a person's sleep and dreams (reviewed in Nielsen and Powell, 2015). It is not uncommon to encounter anecdotes about how eating a certain food or eating too much before bed triggered a bizarre dream or nightmare. In the early twentieth century, cartoonist Winsor McCay published bizarre dreams that the cartoon's protagonists frequently attributed to having eaten Welsh rarebit—a spicy melted cheese toast—or other cheese dishes prior to sleep (Figure 1). However, research relevant to such beliefs is slim, with only a handful of empirical studies having addressed the issue. For example, Kroth et al. (2007), in a survey of 49 participants, found that those who preferred organic foods reported higher dream recall, more recurring dreams, and more dreams containing such themes as flying, whereas participants who preferred fast foods reported lower dream recall and fewer recurring dreams and nightmares. A larger online survey of 436 participants by Biehl (2022) found that dream recall frequency was positively correlated with fruit consumption, that lucid dream recall frequency was positively correlated with fruit, fish, and chili consumption, and nightmare recall frequency was positively correlated with sugary food consumption. However, after partialling out several confounding variables like age and personality, many of these relationships were weakened or eliminated such that no firm conclusions could be drawn.

Whereas the above studies examined the association between food consumption and dreaming, our group (Nielsen and Powell, 2015) conducted a study of 396 undergraduate students that included an assessment of their *perceived* relationship between food and dreaming. We found that 17.8% of students perceived a relationship between what they ate and what they dreamt—which we refer to as “food-dependent dreaming (FDD+)”—with dairy products being the most frequently identified food affecting dreams (39%−44%). Students who perceived food-dependent dreaming also had dreams that were more frequent and disturbing, and they were less likely to rely on feelings of hunger and satiety to determine when to eat. We also found that frequency of disturbing dreams was positively associated with binge-eating and eating for emotional reasons, while frequency of vivid dreams was associated with a healthier diet and longer intervals between meals.

Such findings led us to posit several possible explanations for the common perception that food affects dreaming. These include *food-specific effects* (i.e., certain foods directly affect dreaming), *food-induced distress* (e.g., Lactose Intolerance affecting dreams via gastrointestinal (GI) symptoms), *folklore effects* (e.g., cultural beliefs that foods affect dreams), *misattribution effects* (e.g., non-food influences on dreams, such as stress, that are misattributed to food), and *sleep-effects* (i.e., dietary habits alter sleep and thereby indirectly affect dream content). The latter hypothesis was supported by our finding that the occurrence of disturbing dreams was associated with poorer sleep (Nielsen and Powell, 2015). These findings are also consistent with findings from several other studies, both epidemiological and experimental, that have shown dietary nutrition to affect sleep (see review in Peuhkuri et al., 2012). For example, a survey of 4,435 Japanese workers found associations between dietary habits and insomnia; specifically, low protein intake was associated with difficulties in initiating sleep and poor quality of sleep, and high protein intake and low carbohydrate intake were each associated with difficulties in maintaining sleep (Tanaka et al., 2013). As shown in a recent review (Delage et al., 2024), insomnia symptoms are in turn associated with nightmares, as well as negative dream content and affect. Several laboratory studies have also demonstrated causal influences of food on sleep. For example, a study of 26 healthy adults found that low fiber and high saturated fat and sugar intake were associated with lighter, less restorative sleep with more arousals (St-Onge et al., 2016b). And both poor sleep quality (Paul et al., 2015) and frequent arousals (Blaskovich et al., 2019) are known correlates of frequent nightmares.

In sum, there is a limited, but growing, body of research that supports age-old beliefs that food can influence the content of dreaming and, in particular, the occurrence of nightmares. The present study was designed to shed new light on why dreaming and nightmares are often thought to be food-dependent. Our objective was to test several hypotheses about food's relationship to dreaming, namely, the *food-specific effects, food-induced distress*, and *sleep-effects* hypotheses.

## 2 Methods

### 2.1 Participants

A total of 1,082 participants (721 F; 338 M; 11 non-binary, 2 agender, 3 transgender, 3 gender fluid/queer, 2 not listed, 1 prefer not to disclose, 1 missing) completed an online survey that assessed relationships between eating, sleeping, and dreaming. Data collection took place from January to April 2023 using the Qualtrics Labs, Inc. web platform. Participants were undergraduate students (mean age = 20.3 ± 3.68 yrs old; range: 17–54) enrolled in psychology courses at MacEwan University. Students earned 2% toward their final grade for their participation. Participants identified as White (*n* = 502; 46.4%), Asian (240; 22.2%), Black (85; 7.9%), Arab (68; 6.3%), Native American (16; 1.5%), Latin (14; 1.3%), Hawaiian/Pacific (4; 0.4%), mixed race or “other” (141; 13.0%), or preferred not to answer (12; 1.1%). Of the 970 participants who responded to a question about religious affiliation, most identified as non-religious (*n* = 338; 34.8%), Catholic (245; 25.3%), and Muslim (118; 12.2%), followed by Protestant (42; 4.3%), Sikh (40; 4.1%), Hindu (32; 3.3%), Buddhist (21; 2.2%), Aboriginal Spirituality (12; 1.2%), Jewish (1; 10%), or another religious or spiritual group (121; 12.5%). Men and women participants were compared on all demographic measures and found to differ slightly in age, with women being ~6 months younger (20.1 ± 3.51) than men (20.6 ± 3.90; *t* = 1.96, *p* = 0.05). Age was not found to be an important confounder in statistical analyses; however, gender differences were common, and gender was therefore controlled as a covariable in these analyses.

The study was approved by MacEwan University's Research Ethics Board (ID: 102049). All participants provided informed consent through electronic signature prior to starting the study. They were free to leave blank any questions they did not want to answer and to withdraw from the study at any time without penalty.

### 2.2 Survey

Our online Food & Dreams Survey consisted of both validated and in-house questionnaires that assessed demographic information, dietary habits, health status, personality traits, perceived food-dependent sleep and dreaming, and various sleep and dreaming characteristics, including sleep quality, dream frequency, lucid dreaming, and nightmares.

The measures reported below are a subset of a larger battery of measures in the survey, many of which are not described here.

#### 2.2.1 Demographic information

Information collected included gender, age, ethnicity, religion, medical conditions, psychiatric conditions, current medications, and pregnancy. Due to low numbers of gender diverse participants, only men and women were included in analyses where gender was considered.

#### 2.2.2 Sleep and dreaming characteristics

##### 2.2.2.1 General sleep quality (PSQI-M, PSQI-Q9)

Two measures were derived from the Pittsburgh Sleep Quality Index (PSQI; Buysse et al., 1989) in order to separate measures of sleep quality from those of nightmares, thus allowing their relationships to food and diet to be assessed separately. First, the PSQI modified total score (PSQI-M) was calculated as the sum of all PSQI components except item #9 (*During the past month, how often have you had trouble sleeping because you have bad dreams?*). Also, due to problems in calculating bed- and rise-times (participants estimate these times only to the nearest half-hour), the sleep efficiency component was excluded from this calculation. Second, the PSQI item #9 alone (PSQI-Q9) was used as a supplementary measure of nightmares.

##### 2.2.2.2 Nightmare disorder index (NDI)

Nightmares were assessed with a modified version of the NDI (Dietch et al., 2020). Only 4 of the 5 NDI items were included in the survey, specifically, those which assessed nightmare frequency per week, level of distress, impairment of daily activities, and duration across months. Item 2, which measures frequency of waking up alert from nightmares, was excluded. The definition of NDI thereby assessed only the occurrence of nightmares defined as *disturbing, extended, well-remembered dreams*. Each of the four retained items was scored on 1–5 scales, with total scores calculated as the average of these four items. For comparability with the dream recall measures, a separate item assessed frequency of nightmare recall over the past month (*1: never; 2: <1/mo; 3: about 1/mo; 4: 2–3/mo; 5: about 1/wk; 6: several/wk; 7: almost every morning*).

##### 2.2.2.3 Dreaming, dream emotional tone

One item assessed frequency of dream recall over the past month (*1: never; 2: <1/mo; 3: about 1/mo; 4: 2–3/mo; 5: about 1/wk; 6: several/wk; 7: almost every morning*) and one assessed the average emotional tone of dreams over the past month (*1: very negative; 2: somewhat negative; 3: neutral; 4: somewhat positive; 5: very positive*).

#### 2.2.3 Perceived relationships between food, sleep, and dreams

##### 2.2.3.1 Perceived food-dependent dreaming (FDD) questionnaire

This questionnaire was adapted from the FDD questionnaire used in our previous study (Nielsen and Powell, 2015). It assesses the perceived effects of food on sleep and dreaming. The original FDD questionnaire used three binary (yes/no) and three open-ended questions to determine whether participants had ever noticed that foods led to disturbing or bizarre dreams, or if eating late at night had ever affected their sleep. In the present study, we added questions about whether participants had noticed that eating certain foods or eating late at night led to (1) sleep being better or worse, and (2) dreams being more pleasant, more vivid, more lucid, more bizarre, or more disturbing. If they answered yes to any of these items, they were asked to specify which foods were implicated from a comprehensive list (which included an “other” category). Responses to these questions were collapsed into 2 binary measures assessing whether participants noticed that eating certain foods or eating late at night affected (1) their sleep (=1 if a positive response was given to any of the sleep measures; *otherwise* = 0) and (2) their dreams (=1 or FDD+ if a positive response was given to any of the dreams measures; *otherwise* = 0 or FDD–).

#### 2.2.4 Dietary habits and health status

##### 2.2.4.1 Overall health status (OHS)

General health status was rated with a single item (*How would you rate your overall health status?*) on a 7-point scale from 1: very poor to 7: excellent. Variations of this scale have been shown to be a valid means of assessing a person's general health status (DeSalvo et al., 2006).

##### 2.2.4.2 Patient health questionnaire-4 (PHQ4)

The PHQ4 (Kroenke et al., 2009) is a 4-item self-report questionnaire that assesses depression and anxiety symptoms in the general population. It consists of a 2-item depression scale (PHQ-2) and a 2-item anxiety scale (GAD-2) for which items are rated on 4-point scales (1: not at all; 2: several days; 3: >half the days; 4: nearly every day) in response to the prompt: *Over the last 2 weeks, how often have you been bothered by the following problems?* Validation work on over 5,000 participants (Lowe et al., 2010) justify our use of a single PHQ4 sum score as a pathology indicator. Total scores ranged from 4 to 16, which can be classified as: *normal* (4–6), *mild* (7–9), *moderate* (10–12), or *severe* (13–16; Kroenke et al., 2009). Total scores were used in subsequent analyses.

##### 2.2.4.3 Intuitive eating scale-2 (IES-2)

The IES-2 is a 23-item scale that measures an individual's tendency to follow their physical hunger and satiety cues when deciding when, what, and how much to eat (Tylka and Kroon Van Diest, 2013). The IES-2 Total Scale and four IES-2 subscales [*Unconditional Permission to Eat, Eating for Physical Rather than Emotional Reasons, Reliance on Hunger and Satiety Cues* (in determining when to eat), and *Body-Food Choice Congruence* (in selecting foods that are good for the body)] were used as dependent measures in all analyses.

##### 2.2.4.4 Food sensitivities

Food sensitivities were assessed with eight items. General food intolerance was assessed with a 4-choice item that asked “*Do you have any food intolerances*? (responses: *No, Dairy/Lactose, Gluten, Other - please specify)*.” General Food Allergy was assessed with a binary item asking “*Do you have any food allergies? (No, Yes—please specify)*.” These items were manually screened, and each participant classified as positive or negative for intolerances and allergies. Intolerances were classified as either: (1) *positive for Lactose (but not Gluten) or* (2) *positive for Gluten (but not Lactose)*. Instances of *positive for both Lactose and Gluten, negative for both, or positive for an Unspecified intolerance* were very rare and excluded from analysis. Allergies were classified as positive for either *Fruit, Shellfish/Fin-fish, Peanut/Tree-nut* or *Other*, with responses summed to create a *Food Allergy* score that ranged from 0 to 4.

##### 2.2.4.5 Gastrointestinal symptoms questionnaire (GISQ)

We administered the GISQ (Bovenschen et al., 2006) on which participants rated how often they experience six separate GI or headache symptoms on 5-point Likert scales (0: never; 1: occasionally; 2: sometimes; 3: often/most days; 4: always/almost always). They then rated how severe those symptoms were on six 11-point Likert scales (0: no symptoms to 10: max symptoms). To reduce the overall number of measures, we computed two subscale scores that separately summed frequencies and severities for the six items and a 3rd subscale that averaged the products of frequency × severity scores for the six items. The latter composite score (GI-FxS) was used in subsequent analyses.

##### 2.2.4.6 Eating in the absence of hunger (EAH)

EAH is a three-item measure of overeating that asks “*Do you ever eat large amounts of food when you don't feel hungry?*” “*Do you ever eat a snack when you don't feel hungry?*” *and* “*Do you ever eat a meal when you don't feel hungry?*” which has been shown to be a significant predictor of executive dysfunction (Powell and Klaver, 2022). Responses using 5-point Likert scales (1: never or almost never to 5: always or almost always) were summed to produce a total EAH score ranging from 3 to 15.

##### 2.2.4.7 Healthy eating assessment (HEA)

The HEA from the Government of Northwest Territories (adapted from Paxton et al., 2011) was used to identify general eating patterns and levels of healthy eating. Although our original assessment included 10 items with 1–5 Likert scales to assess *daily* intakes, to reduce the overall number of measures we opted to use only item 1 (*HEA1: How would you rate your overall habits of eating healthy foods?*) as a general measure of healthy eating.

##### 2.2.4.8 Night eating questionnaire (NEQ)

The NEQ is a 14-item scale that assesses the behavioral and psychological symptoms of Night Eating Syndrome (Allison et al., 2008). Two scale items that assessed depressed mood were omitted, as were six items that assessed impact of night-eating. Principal components factor analyses of the remaining eight items revealed a 2-factor solution that accounted for 60.9% of the variance and included a clear Night-eating factor (39.0% VAF: *Do you have cravings or urges to eat snacks when you wake up at night? Do you need to eat in order to get back to sleep when you awake at night? When you get up in the middle of the night, how often do you snack? How many times per week did you wake up in the middle of the night to eat something?*) and a clear Evening-eating factor (21.9% VAF: *Do you have cravings or urges to eat snacks after supper, but before bedtime? How much control do you have over your eating between supper and bedtime? How much of your daily food intake do you consume after supper? How many times per week did you eat a snack in the evening (after supper but before sleep?))*. These two sets of factor scores were used in subsequent analyses. Two in-house questions were also added to assess the frequency of evening-eating and night-eating over the last month.

### 2.3 Statistical analyses

Analyses were conducted using SPSS 26 for Windows. Data reduction for multi-item questions was achieved using factor analysis with principal components extraction, Kaiser normalization and Varimax (factor-orthogonal) rotation. Regression analyses were achieved with linear regression. Spearman correlations were used when response distributions were non-Gaussian in nature. *P*-values of α = 0.05 were used to indicate statistical significance for regressions, correlations, chi-squares, *t*-tests (2-tailed) and mediation analyses. The mediation analyses were conducted using the Structural Equation Modeling (SEM) function in R, with standard errors obtained via bootstrapping with 5,000 samples and standardized coefficients calculated. Results were displayed with GraphPad Prism 9.

## 3 Results

### 3.1 General sleep, dream, dietary, and health characteristics

#### 3.1.1 Dreaming and nightmare measures

Dream and nightmare recall frequencies over the last month for the entire sample are shown in Supplementary Table S1. Overall, 32.2% (347/1,078) of participants had a clinically significant frequency of recalled nightmares (i.e., 1+/week). Also shown in Supplementary Table S1, all measures differed by gender in that women recalled more dreams and more nightmares, had dream emotion that was on average more negative, and scored higher on the NDI and the PSQI-Q9 (all *p*'s < 0.001). Finally, the proportion of women having at least 1 nightmare/week was higher (36.6%) than it was for men (20.8%; χ^2^ = 26.28, *p* < 0.001).

#### 3.1.2 General health

Overall health status was rated to be 4.91 ± 1.17 or almost 1 point above the midpoint of the 1–7 scale. Women rated their overall health status as lower (4.80 ± 1.17) than did men (5.20 ± 1.11; *t*_953_ = 4.98, *p* < 0.001). On the item assessing any medical condition, 13.8% of participants responded yes, 81.7% reported no, and 4.5% preferred not to disclose. For the item assessing any psychiatric condition, 17.1% reported yes, 80.1% reported no, and 2.8% preferred not to disclose. Taken together, 25.8% (265/1,029) of participants reported having at least 1 medical or psychiatric condition, with more women (31%) reporting a condition than men (9%; *X*^2^ = 38.86, *p* < 0.001). Of the 1,077 participants responding to the question about taking medications on a daily or near-daily basis, 270 (25.1%) said yes, 766 (71.1%) said no, and 41 (3.8%) preferred not to disclose. Again, more women (31%) than men (9%) reported taking medications (*X*^2^ = 67.89, *p* < 0.001). Overall score on the PHQ4 was 9.50 ± 3.48 which falls at the border of *mild* (7–9) and *moderate* (10–12) pathology (Kroenke et al., 2009); scores were higher for women (9.98 ± 3.41) than for men (8.38 ± 3.37; *t*_1054_ = 7.61, *p* < 0.001). Four of the participants (0.4%) reported being pregnant.

#### 3.1.3 Perceived influence of food on sleep quality

Percentages of participants who named foods that produce either better or worse sleep are shown in Supplementary Table S2.

#### 3.1.4 Food sensitivities (intolerances/allergies)

A total of 32.4% (351/1,082) of participants reported some form of food sensitivity. This was true for almost twice as many women (38.1%) as men (19.8%; χ^2^ = 35.32, *p* < 0.001). Similar gender differences were found for Food Allergy (*p* = 0.004), Lactose Intolerance (*p* < 0.001), and Gluten Intolerance (*p* = 0.013). Gender was used as a covariable when assessing further relationships between food sensitivities and sleep or dreams.

### 3.2 Perceived influence of food on sleep and dreams

Many participants reported that eating affected their sleep in some way: 435 (40.2%) said that their sleep was either improved or worsened either by eating particular foods (374 or 34.6%) or by eating late (137 or 12.7%). A total of 267 (or 24.7%) of participants claimed that eating particular foods made their sleep worse while 218 (or 20.1%) claimed that foods made it better. As shown in Supplementary Table S2, the majority of participants indicated that better sleep was induced by Fruit (17.6%), Herbal Tea (13.4%), and Vegetables (11.8%), which were also among the least often mentioned instigators of worse sleep (1.7%−3.2%). Participants blamed worse sleep mainly on Desserts/Sweets (22.7%), Spicy food (19.5%), and Dairy (15.7%).

A total of 59 (or 5.5%) of 1076 participants responding reported that foods or eating late affected their dreaming; we refer to such reports as food-dependent dreaming (FDD+). Thirty-five (of 1070) participants (3.3%) reported that eating late affected their dreams (replies of No: 656 or 61.3%; Unsure: 379 or 35.4%). FDD+ was marginally higher in women (46 or 6.4%) than men (12 or 3.6%; χ^2^ = 3.56, *p* = 0.061). FDD+ participants reported a higher score than FDD- participants on the NDI (3.61 ± 3.18 vs. 2.43 ± 3.03; *t*_1073_ = 2.90, *p* = 0.004) which was due entirely to a higher nightmare frequency (1.19 ± 0.90 vs. 0.81 ± 0.85; *t*_1073_ = −3.29, *p* = 0.001).

To test the *food-specific effect* hypothesis, we asked FDD+ participants whether specific food categories affected their dreams. FDD+ participants claimed that foods caused their dreams to become more vivid (*n* = 30/55 or 54.5%), more pleasant (11/46 or 23.9%), more disturbing (27/47 or 57.4%), more bizarre (30/50 or 60.0%), or more lucid (7/47 or 14.9%). Further, as shown in Table 1, most of the choices blamed for dream changes were Desserts/Sweets (30%), Dairy (e.g., milk, yogurt, cheese, other; 21%), Fruit (9%), Meat (excluding Seafood; 8%), and Cereals/Grains (bread, pasta, rice, etc.; 6%). All other categories were blamed <6% of the time. Pleasant dream changes were blamed on a relatively even distribution of food categories, including Desserts/Sweets (11%), Dairy (11%), Fruit (15%), Vegetables (11%), and Herb Tea (11%); disturbing dream changes, however, were blamed primarily on Desserts/Sweets (31%) and Dairy (22%), and secondarily on Meat (16%) and Spicy foods (13%).

**Table 1:** Number of times each of 14 food groups was named as causing dreams to become more vivid, disturbing, bizarre, pleasant, or lucid.

**Food group**	**Vivid**	**%**	**Pleasant**	**%**	**Disturbing**	**%**	**Bizarre**	**%**	**Lucid**	**%**	**Total**	**%**
Desserts/Sweets	13	33	3	11	10	31	10	38	3	50	39	29.8
Dairy	9	23	3	11	7	22	7	27	1	17	27	20.6
Fruit	5	13	4	15	1	3	2	8	0	0	12	9.2
Meat	1	3	2	7	5	16	2	8	1	17	11	8.4
Cereals	3	8	2	7	1	3	2	8	0	0	8	6.1
Spicy foods	1	3	0	0	4	13	2	8	0	0	7	5.3
Vegetables	3	8	3	11	0	0	0	0	0	0	6	4.6
Herb tea	3	8	3	11	0	0	0	0	0	0	6	4.6
Non-Caf beverages	1	3	1	4	1	3	1	4	1	17	5	3.8
Eggs	1	3	2	7	0	0	0	0	0	0	3	2.3
Seafood	0	0	1	4	2	6	0	0	0	0	3	2.3
Nuts	0	0	1	4	1	3	0	0	0	0	2	1.5
Veg protein	0	0	1	4	0	0	0	0	0	0	1	0.8
Pickled	0	0	1	4	0	0	0	0	0	0	1	0.8
**Total food**	40		27		32		26		6		131	100

Alcohol, Caffeine, Vitamins, Other, and Unspecified food groups were excluded. Dream category percentages can be > 100 for some categories because participants could select more than one food group for each dream category. See text for label definitions.

### 3.3 Dream and sleep relationships to food sensitivities

To test the *food-induced distress* and *sleep-effects hypotheses*, we next assessed whether allergies and intolerances were related to the belief that food affects dreaming or sleep. As shown in Table 2A, FDD+ participants were 2× more likely to have Food Allergy and 3× more likely to have Gluten Intolerance, but not more likely to have Lactose Intolerance.

**Table 2:** Prevalence of food sensitivities for participants who perceive (Yes/No) that food leads to altered dreaming, better sleep, or worse sleep.

**Food sensitivity**	**% Yes**	**% No**	**χ^2^**	***p***
**A. Food affects dreaming (FDD)?**				
Food Allergy	18.6	9.3	5.43	0.039
Gluten Intolerance	6.8	2.1	5.46	0.043
Lactose Intolerance	28.8	21.6	1.67	0.198
**B. Food causes better sleep?**				
Food Allergy	11.0	9.6	0.38	0.527
Gluten Intolerance	2.8	2.3	0.14	0.629
Lactose Intolerance	22.9	21.8	0.14	0.715
**C. Food causes worse sleep?**				
Food Allergy	12.7	9.0	3.22	0.077
Gluten Intolerance	4.1	1.8	4.46	0.062
Lactose Intolerance	29.6	19.5	11.91	<0.001

As shown in Table 2B, participants who did and did not report that food *improved* their sleep did not differ in any food sensitivities. On the other hand, participants who reported that food *worsened* their sleep were more likely to report Lactose Intolerance (*p* < 0.001; Table 2C).

The findings for worse sleep were confirmed by assessing self-rated sleep quality on the PSQI-M. The presence (vs. absence) of Lactose Intolerance was associated with lower sleep quality as indexed by a higher PSQI-M score (7.76 ± 2.90 vs. 6.90 ± 2.80; *t*_1080_ = 4.14, *p* < 0.001).

In sum, participants with food allergies and Gluten Intolerance were more likely to report that food affects dreaming, while those with Lactose Intolerance were more likely to report that food causes worse sleep.

### 3.4 Dream/nightmare correlates of healthy eating and food sensitivities

To assess relationships between dreaming and healthy eating habits, stepwise multiple regression analyses were conducted using each of three dreaming indicators (*dream recall, dream emotional tone*, and *NDI*) as dependent measures, two blocks of control variables, and a 3rd block of diet-related independent variables, i.e., Block 1: Gender; Block 2: mental health (PHQ4), sleep quality (PSQI-M), any medical/psychiatric condition, and any daily/near-daily medications; Block 3: GI symptoms (GI-FxS), eating in the absence of hunger (EAH), overall health status (OHS), intuitive eating subscales 1–4 (IES1–IES4), Night-eating/Evening eating factor scores, and Healthy Eating Assessment Item 1 (HEA1). Nightmare recall (NMR) was added to Block 2 for assessments of dependent measures *dream recall* and *dream emotional tone* whereas dream recall (DR) was added to Block 2 for assessment of *NDI*. Gender was controlled as this variable was found to differ on several of the dependent and independent measures. These analyses produced the following three models:

- *Dream Recall*: higher recall was predicted by higher Body-Food Congruence subscale scores on the IES (Standardized β = 0.062, *t* = 2.32, *p* = 0.022), less Evening-Eating (β = −0.110, *t* = −4.06, *p* < 0.001), and fewer anxiety/depression symptoms as assessed by the PHQ4 (β = −0.074, *t* = −2.45, *p* = 0.019; total *R* = 0.584; adjusted VAF = 0.337, *F*_6,954_ = 82.32, *p* < 0.001);
- *Emotional Tone of Dreams*: negative tone was predicted by more GI symptoms on the GI-FxS (β = 0.087, *t* = 2.79, *p* = 0.006), less reliance on Hunger and Satiety Cues on the IES (β = −0.065, *t* = −2.16, *p* = 0.027), higher anxiety/depression scores on the PHQ4 (β = 0.165, *t* = 5.29, *p* < 0.001), and more medication use (β = 0.085, *t* = 2.81, *p* = 0.005; total *R* = 0.434; adjusted VAF = 0.183, *F*_6,954_=36.94, *p* < 0.001);
- *Nightmare Disorder Index (NDI):* higher scores were predicted by more GI symptoms on the GI-FxS (β = 0.140, *t* = 4.76, *p* < 0.001), higher Evening-Eating scores (β = 0.197, *t* = 6.98, *p* < 001), the presence of any medical/psychiatric condition (β = 0.130, *t* = 4.42, *p* < 0.001), and higher PHQ4 scores (β = 0.167, *t* = 5.57, *p* < 0.001; total *R* = 0.498; adjusted VAF = 0.243, *F*_6,954_ = 52.34, *p* < 0.001).

In sum, regression analyses revealed relationships between eating habits and both dream recall and dream negativity, independent of gender and other pathological factors. While dream recall was predicted by healthy eating, as reflected in a high body-food congruence and less evening eating, dream negativity was predicted by three indicators of unhealthy eating: GI symptom severity, more evening eating, and lower reliance on hunger and satiety cues.

**Figure 2:**
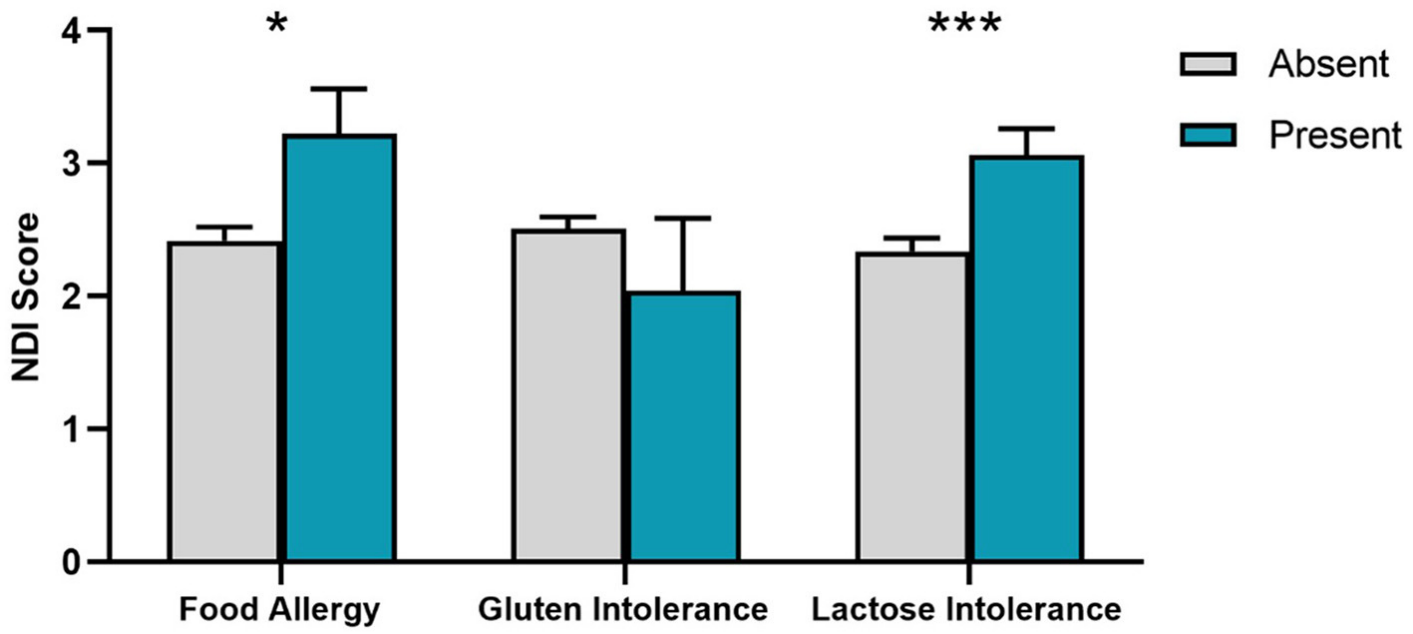
Nightmare Disorder Index (NDI) scores for presence/absence of food sensitivities. SEM error bars are shown. *p < 0.05; ***p < 0.001.

We next assessed whether food sensitivities were associated with dreaming and nightmares using group comparisons. As shown in Figure 2, participants with Food Allergy had higher NDI scores than those without Food Allergy (*t*_123.4_ = 2.29, *p* = 0.012). This was also the case for participants reporting Lactose Intolerance (*t*_1079_ = 3.26, *p* < 0.001), but not for Gluten Intolerance (*t*_1079_ = 0.22, *p* = 0.436).

To explore possible graded relationships between dreaming and food sensitivities, three separate multiple regressions were conducted using *dream recall, dream emotional tone* and *NDI* scores as dependent measures, gender as a covariate and the three food sensitivity measures (*Food Allergy, Gluten Intolerance, Lactose Intolerance*) as predictors. These analyses found:

- *Dream Recall*: no effects for dream recall other than Gender (β = −0.145, *t* = −4.77, *p* < 0.001);
- *Emotional Tone of Dreams*: no effects for dream emotional tone other than Gender (β = 0.123, *t* = 4.021, *p* < 0.001);
- *Nightmare Disorder Index (NDI)*: Lactose Intolerance (β = 0.074, *t* = 2.43, *p* = 0.015) and Food Allergy (β = 0.069, *t* = 2.26, *p* = 0.024) both independently predicted NDI. Holding constant GI symptoms (GI-FxS) eliminated the Lactose Intolerance effect (β = 0.037, *t* = 1.22, *p* = 0.221) but not the Food Allergy effect (β = 0.065, *t* = 2.19, *p* = 0.028). Similarly, holding constant depression and anxiety symptoms (PHQ4) diminished the Lactose Intolerance effect (β = 0.056, *t* = 1.88, *p* = 0.060) but not the Food Allergy effect (β = 0.073, *t* = 2.45, *p* = 0.014). Finally, holding constant sleep quality (PSQI-M) did not diminish the independent contributions of Lactose Intolerance (β = 0.066, *t* = 2.13, *p* = 0.033) or Food Allergy (β = 0.064, *t* = 2.12, *p* = 0.034) to NDI scores, even though the rating of general sleep quality was associated with NDI scores (β = 0.088, *t* = 2.88, *p* = 0.004).

**Figure 3:**
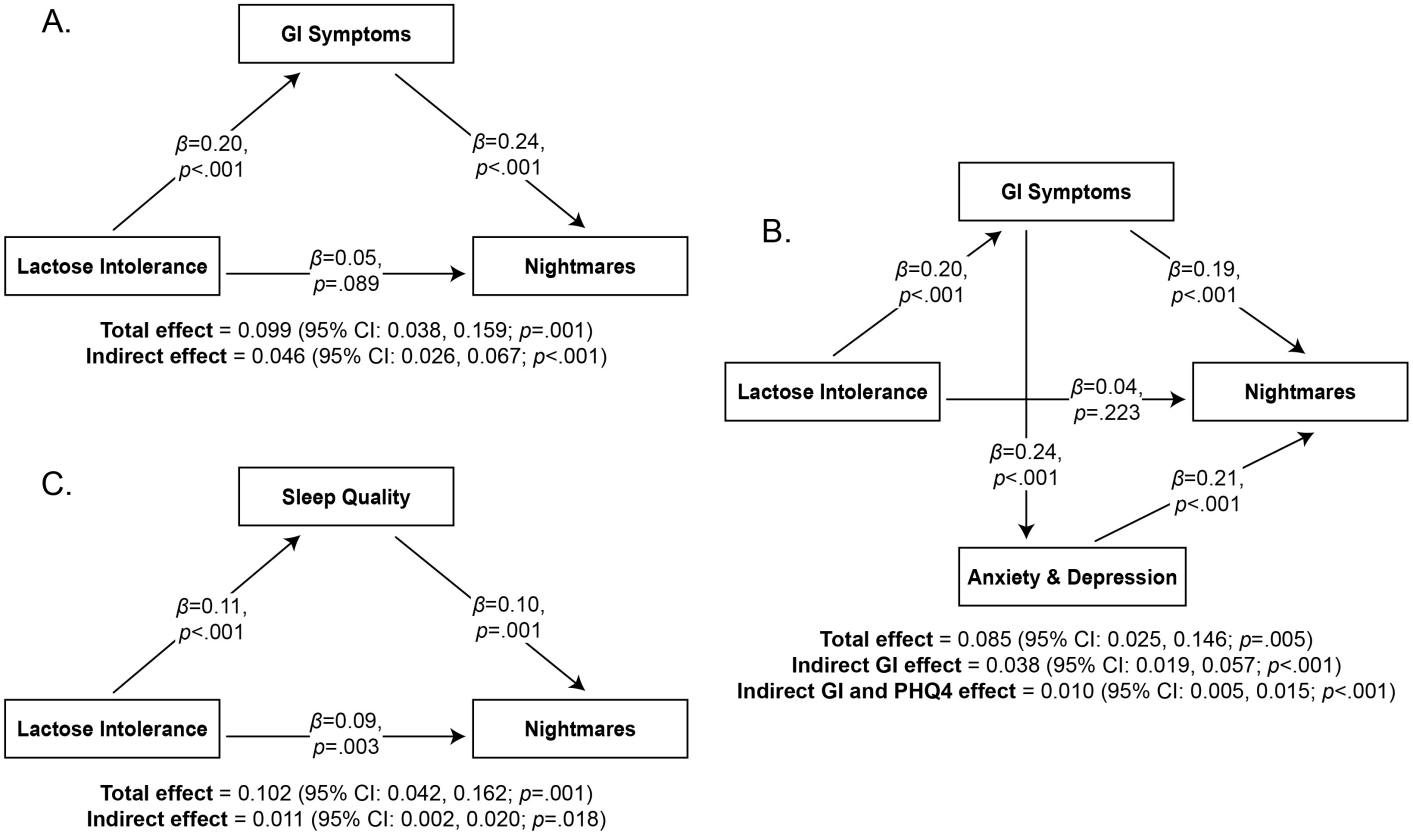
Mediation analyses of the relationship between Lactose Intolerance and Nightmares with standardized beta coefficients. The mediating variables are GI Symptoms (Panel **A**), both GI Symptoms and Anxiety/Depression (Panel **B**), and Sleep Quality (Panel **C**).

To assess whether GI symptoms mediated the relationship between Lactose Intolerance and NDI, we conducted a mediation analysis using Structural Equation Modeling (SEM; Figure 3). In the model (4,984 bootstrap samples), the independent variable was Lactose Intolerance, the mediating variable was GI symptoms, and the dependent variable was nightmare severity (NDI). The model was just-identified, meaning that there were 0 degrees of freedom and typical tests of model fit were not applicable (e.g., CFI = 1.00, TLI = 1.00, RMSEA = 0.00, SRMR = 0.00). The analysis therefore focused on the interpretation of the parameter estimates.

It revealed a significant indirect effect of Lactose Intolerance on nightmares through GI symptoms (β = 0.046, 95% confidence interval = 0.026, 0.067; *p* < 0.001; Figure 3A). An additional exploratory analysis was performed to determine if anxiety and depression symptoms (PHQ4) play a role in these relationships (Figure 3B). This model (4,999 bootstrap samples) followed the same structure and added PHQ4 as a mediating variable between GI symptoms and NDI. It examined two indirect effects: (1) Lactose Intolerance through GI symptoms on NDI, and (2) Lactose Intolerance through GI symptoms through PHQ4 on NDI. Most of the model scores indicated good fit (CFI = 0.986, TLI = 0.918, RMSEA = 0.053, SRMR = 0.019). Results replicated the previous model in that there was a significant indirect effect of Lactose Intolerance on NDI through GI symptoms (β = 0.038, 95% confidence interval = 0.019, 0.057; *p* < 0.001). It also revealed a significant indirect effect of Lactose Intolerance on nightmares through GI symptoms through PHQ4 (β = 0.010; 95% confidence interval = 0.005, 0.015; *p* < 0.001). These findings suggest that the mediating effect of GI symptoms on NDI can be partially explained by associated anxiety and depression symptoms.

Finally, to further test the *sleep-effects* hypothesis, we ran two additional mediation analyses to determine whether sleep quality (PSQI-M) plays a role in this relationship. In the first model (4,982 bootstrap samples; Figure 3C), Lactose Intolerance was the independent variable, sleep quality (PSQI-M) the mediating variable, and nightmare severity (NDI) the dependent variable. This model was also just-identified; it revealed a significant indirect effect of Lactose Intolerance on NDI through sleep quality (β = 0.011; 95% confidence interval = 0.002, 0.020; *p* = 0.018). In the second model (5,000 bootstrap samples), Lactose Intolerance was the independent variable, GI symptoms the first mediating variable, sleep quality the second mediating variable, and NDI the dependent variable (not shown). Most model scores indicated good fit (CFI = 0.974, TLI = 0.847, RMSEA = 0.063, SRMR = 0.021). This analysis replicated the first and second analyses in that a significant indirect effect of Lactose Intolerance on NDI through GI symptoms was found (β = 0.044; 95% confidence interval = 0.023, 0.065; *p* < 0.001). However, the indirect effect of Lactose Intolerance on NDI through GI symptoms through sleep quality was not significant (β = 0.002; 95% confidence interval = −0.001, 0.005; *p* = 0.117). These two mediation analyses suggest that Lactose Intolerance may decrease sleep quality through additional mechanisms than GI symptoms, and thereby contribute to nightmares.

In sum, GI symptoms consistently and significantly mediated relationships between Lactose Intolerance and nightmares. One possible trajectory is that GI symptoms increase nightmares by increasing symptoms of anxiety and depression. Sleep quality also partially mediated the relationship between Lactose Intolerance and nightmares. However, the indirect effect of Lactose Intolerance on nightmares through GI symptoms and sleep quality was not significant, suggesting that variables other than GI symptoms may be important for explaining the relationship between Lactose Intolerance, sleep quality, and nightmares.

### 3.5 Correlates of night- and evening-eating

Having found general relationships between Lactose Intolerance, sleep, and nightmares, we looked more closely at the association of eating at night (Night-Eating) and eating close to bedtime (Evening-Eating) with sleep and dreaming measures. As shown in Table 3, Night- and Evening-Eating were both associated with poor sleep on the PSQI-M (both *p*'s < 0.001), more negative emotional tone of dream content (*p* < 0.001 and *p* = 0.029, respectively) and higher NDI scores (both *p*'s < 0.001). Neither factor score was associated with dream recall (both *p*'s > 0.233).

**Table 3:** Spearman correlations (2-tailed) between PSQI-M, PSQI-Q9, dream and nightmare measures, and night- and evening-eating factor scores.

**Sleep/dream measure**	**Night-eating**	**Evening-eating**
Sleep quality (PSQI-M)	0.115^***^	0.156^***^
Dream recall frequency (DRF)	0.017	−0.036
Dream emotional tone	−0.120^***^	−0.067^*^
Nightmare disorder index (NDI)	0.126^***^	0.121^***^

^*^p < 0.05; ^***^p < 0.001.

## 4 Discussion

### 4.1 Proposed hypotheses about food and dreaming

Our findings provide evidence that both supports and refutes the hypotheses we proposed to possibly explain relationships between food and dreaming (Nielsen and Powell, 2015).

#### 4.1.1 Food-specific effects hypothesis

Some of the present findings are pertinent to the *food-specific effects hypothesis* which stipulates that participants who report food-dependent dreaming are noticing cause-and-effect relationships between specific foods and the nature of their dreams. Such participants may be more sensitive to dreaming and have a greater sensitivity to detecting the effects of certain foods. In the present findings, participants identified some foods that they believed to influence their dreams and their sleep; desserts/sweets and dairy were particularly noteworthy in that they were most often identified as affecting both dreaming (29.8% and 20.6%, respectively) and the worsening of sleep (22.7% and 15.7%). In contrast, fruits (17.6%), vegetables (11.8%), and herbal teas (13.4%) were most often identified as leading to better sleep. While we still lack substantive evidence that these participant observations for food and dreaming are, in fact, accurate, there is some suggestive evidence for their accuracy. For example, one study (Noorwali et al., 2018) found that both short sleepers (< 7 h/night) and long sleepers (>8 h/night) had lower overall intakes of fruits and vegetables than did those who slept a more normal 7–8 h per night. Other studies have reported associations between short or poor-quality sleep and the consumption of sugary foods (Katagiri et al., 2014; Shahdadian et al., 2023) or between better quality sleep and the daily consumption of specific foods like kiwis, tart cherry juice, or fatty fish [see (St-Onge et al., 2016a) for review]. For example, a tart cherry juice beverage improved some insomnia symptoms relative to a placebo beverage (Pigeon et al., 2010). Such findings are consistent with the claim that some participants are able to accurately identify foods that affect sleep—and, by extension, dreaming.

With respect to participants' reports of food-dependent dreaming, there was a much lower prevalence of FDD+ participants in the present cohort (5.5%) than in our previous one (17.8%; Nielsen and Powell, 2015). This was the case even though the present participants were provided with more probes about such effects than were our previous participants. This lower prevalence suggests that, if food-specific effects exist in this cohort, they were noticed by relatively few people. The large discrepancy in prevalence between our two studies is perhaps surprising in that the participant cohorts were drawn from the same first-year psychology courses offered at the same University and had roughly the same ages (21.4 ± 5.15 vs. 20.3 ± 3.68). However, the discrepancy might be partially explained by the possibility that the considerable time difference (~11 years) separating the two cohorts may have caused them to differ in several important respects. One possibility, which our survey did not address in detail, is that participants in the present cohort were more aware of good food hygiene practices than were the participants in previous cohort. Another possibility is related to the fact that medical and public awareness of food sensitivities—Lactose Intolerance in particular—increased markedly during this period of time; for example, in Canada while there was an increase (+2.3%) in all perceived food allergies combined from 2010 to 2016, the increase in milk allergy was greatest (+1.9%), affecting both children (<18 y; +1.2%) and adults (+2.1%; Clarke et al., 2020). Thus, many people in the present cohort may have been more cognizant of food hygiene and their own food sensitivities, may have more successfully avoided culprit foods, and may thereby have had less experience with their dreams being influenced by such foods. Second, the present cohort recently lived through the COVID-19 pandemic and experienced a range of profound—and sometimes paradoxical—pandemic-related effects. The latter included not only intensification of their dreams and nightmares, but also a clear focus of dream content on pandemic themes (Gorgoni et al., 2022; Margherita and Caffieri, 2023; Parrello and Sommantico, 2022). There was likewise an increase in sleep disorders, disruptions of normal sleep routines, and aggravation of mental health symptoms (Valenzuela et al., 2023; Windarwati et al., 2022). These changes were especially true for high school students (Windarwati et al., 2022) which a majority of the participants in the present study would have been at the time. Perhaps most surprisingly, while the pandemic produced an *improvement* in nutrition (Aksoy et al., 2021; Hernandez-Nava et al., 2022), it was also associated with an increase in nightmares (Hernandez-Nava et al., 2022). Finally, other societal changes over the last decade, such as a marked increase in the use of social media (Aksoy et al., 2021), generational differences in cultural myths, or less intergenerational passing on of notions about food affecting dreams, could also have contributed to an increased awareness of food sensitivities, to a decreased awareness or interest in dream experience, or to changes in other related factors. Altogether, such factors may underlie a generational shift in awareness of food's relationship with dreaming.

Thus, it is possible that awareness of food sensitivities, pandemic-induced fears and preoccupations combined with other cultural and societal changes somehow led to a net diminishment in our participants' abilities to perceive food-dependent dreaming. Further study of a number of these factors is clearly warranted.

#### 4.1.2 Food distress hypothesis

The *food distress hypothesis* suggests that perceptions of food-dependent dreaming result from the effects of food allergies and intolerances, the symptoms of which induce noticeably bizarre and disturbing dreams. The ‘Rarebit Fiend' version of this hypothesis, i.e., blaming cheesy meals in particular, was supported in the present results by (1) our participants' perceptions that dairy products were the 2nd most prevalent food group responsible for affecting their dreams—including disturbing (22%) and bizarre (27%) dreams—and (2) the finding that nightmares were, in fact, robustly associated with Lactose Intolerance. These observations extend our previous finding that dairy products were the most often cited culprits of altered dreaming (Nielsen and Powell, 2015). Together, these results form a basis for suggesting that the associations between dairy consumption and disturbing dreams—like those portrayed in the Dreams of the Rarebit Fiend cartoon series—may be due to Lactose Intolerance and its accompanying GI distress.

Two additional findings in the present study suggest that the *food distress hypothesis* may be even more broadly applicable. The fact that nightmares were associated with Food Allergy independent of their relation to Lactose Intolerance, and that food-dependent dreaming was associated with both Food Allergy and Gluten Intolerance suggests that food sensitivities beyond Lactose Intolerance may have effects on dreams. For example, some features of food allergies—the most prominent being the threat of life-threatening reactions—can lead to high levels of emotional distress and anxiety (Polloni and Muraro, 2020) which in turn might affect dream content. Living with food allergies might also make participants more sensitive and attuned to what they eat, which in turn could make them more likely to attribute dream changes to their diet.

**Figure 4:**
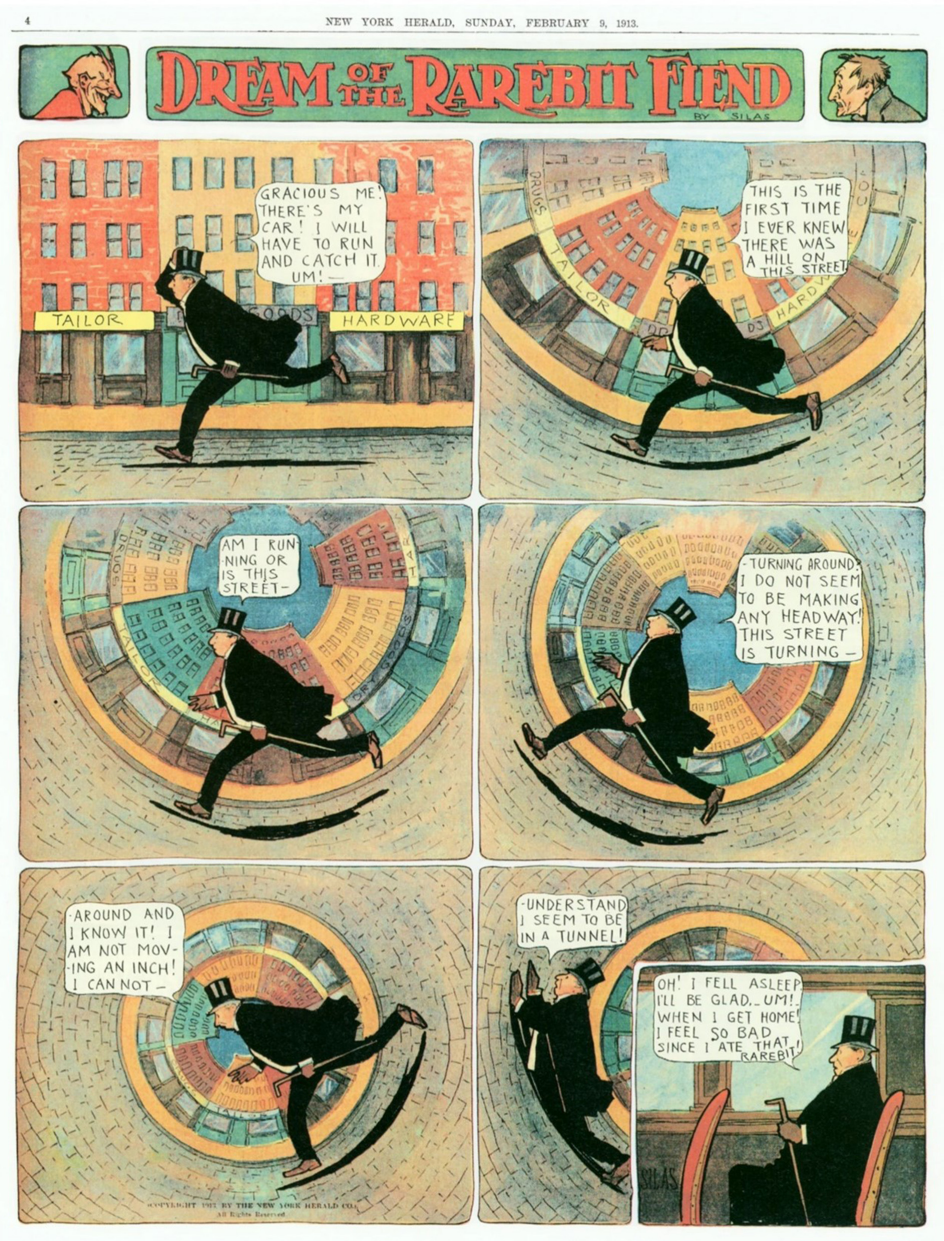
In many of his closing panels, protagonists of the Rarebit Fiend cartoonist Winsor McCay explicitly blamed bizarre events occurring in their dreams and nightmares on symptoms associated with eating cheese dishes prior to sleep. We speculate that these symptoms may have been linked to the artist himself being afflicted with a Lactose Intolerance condition. (Reproduced from “Dream of the Rarebit Fiend” by Windsor McCay, Dream of the Rarebit Fiend by Winsor McCay : Winsor McCay : Free Download, Borrow, and Streaming : Internet Archive, CC0).

Importantly, we also found a significant indirect effect of GI symptoms on the relationship between Lactose Intolerance and nightmares. This analysis supports the notion that specific food-induced symptoms such as bloating, cramping or excess gas arising during sleep have a negative impact on dreaming. Such a mediating role for GI symptoms is consistent with other findings that dreaming is more emotionally intense and conflictual when abdominal cramping is at its worst, e.g., during menstruation (see review in Nielsen, 2005). The possibility that GI symptoms can incite nightmares could explain why Winsor McCay so consistently asserted in his art that Welsh rarebit and other cheese snacks so wildly affected dreams (see Figures 1, 4): McCay himself may have had nightmares aggravated by Lactose Intolerance!

In sum, not only do participants perceive relationships to exist between disturbed dreaming and dairy (among other food groups), but such dreaming is associated with a specific sensitivity to dairy, and possibly to a wider variety of allergies and intolerances as well. GI symptoms may be an important factor mediating the association between Lactose Intolerance and nightmares.

#### 4.1.3 Sleep-effects hypothesis

This hypothesis stems from evidence that dietary habits can worsen sleep (Choi et al., 2022; Oliveira and Marques-Vidal, 2023) and that sleep disruption, in turn, is frequently associated with nightmares. Accordingly, diet may incite nightmares by the intermediation of various types of sleep disturbance. Two types of findings in the present results provide support for this hypothesis: (1) evidence that dietary content affects sleep quality and (2) evidence that the timing of meals affects sleep quality.

First, with respect to dietary content, we found that well over a third (40.2%) of participants claimed that eating particular foods affected their sleep, with almost a quarter (24.7%) of them claiming that it worsened their sleep. Some specific foods were named as responsible for such changes, mainly, sweets/desserts, spicy foods and dairy. These participant observations are, in fact, consistent with many studies of diet and sleep quality finding that particular foods are associated with either better (see review in Peuhkuri et al., 2012) or worse (Oliveira and Marques-Vidal, 2023) sleep. For example, fish, vegetables, fruit, and cheese intake are associated with a better sleep quality as measured by the PSQI, while sugar and meat intake are associated with poorer sleep quality (Oliveira and Marques-Vidal, 2023). Findings for all of these food groups except fish were replicated in the present findings. The relevance of these findings for dreaming is that diminished sleep quality is associated with more frequent nightmares and more negatively toned dreams (e.g., Goitom and Schredl, 2020), a finding that we replicate in this study. We also found that self-perceived worse sleep was associated with food sensitivities, to Lactose Intolerance in particular. However, controlling for general sleep quality with the PSQI-M score did not eliminate the above-mentioned relationship between Lactose Intolerance and NDI, nor the relationship between Food Allergy and NDI. Thus, although sleep quality may have been one factor mediating relationships between food and dreaming in the present study, it is clearly not the only one.

Note that the direction of causality in many studies of food and sleep remains unclear. For example, it is unclear if the fact that individuals who sleep less have poorer diets (Peuhkuri et al., 2012) is because poor sleep leads to the adoption of such habits, because a poorer diet leads to poor sleep or because of some 3rd factor influencing both.

Second, with respect to the timing of meals, misalignment of eating times can shift one's chronotype toward an eveningness pattern, and the night-eating of meals is associated with an eveningness chronotype (Kandeger et al., 2021). Eveningness is also robustly associated with nightmares (Munezawa et al., 2011; Nielsen, 2010; Sandman et al., 2016). Congruent with these findings, we found a direct association between night-eating and nightmares. However, because eveningness and night-eating are also both associated with poor sleep quality (Fabbian et al., 2016; Merikanto et al., 2012; Sheaves et al., 2016), it may be speculated that the effect of meal timing on dreaming is not completely independent of dietary content as described in the previous section.

#### 4.1.4 Future directions: misattribution and folklore hypotheses

Although our study was not designed specifically to address the *misattribution* and *folklore hypotheses* about perceived food effects on dreaming, some results are nonetheless pertinent to them and point to future avenues of research. These two hypotheses are considered together here because, in lieu of empirical findings, the misattributed source of a real effect is frequently dictated by folk wisdom. In the present results, a real effect is strongly supported by the findings that nightmares and negative dream emotions are associated with Lactose Intolerance; however, these dream changes may have been attributed by participants to food sensitivities *other than* Lactose Intolerance. In short, FDD+ participants appeared to be misattributing a real link between Lactose Intolerance and negative dreaming to many factors *other than* the food that was likely responsible for the dream changes. Such misattributions about dreaming—especially about negative dreaming—are not rare and are often coupled with folklore-based beliefs. For example, it is believed in many cultures that nightmarish experiences of a menacing presence produced by *Recurrent Isolated Sleep Paralysis* attacks are actually encounters with demonic figures (for review see Olunu et al., 2018). Moreover, scholars in some early societies even misattributed these nightmarish episodes to GI symptoms, such as the rising of *vapors from the stomach to the brain*, and they prescribed changes in diet to treat them (Dannenfeldt, 1986; Golzari et al., 2012; Olunu et al., 2018). Although our findings do not speak directly to such early beliefs—the absence of any direct assessment of participants' culturally informed beliefs is a clear limitation of the study—they do suggest that dietary interventions may be usefully explored as possible treatments for sleep paralysis.

### 4.2 General implications

Insofar as the present findings are essentially correlational in nature, it remains unclear whether the dietary factors assessed cause dream changes, whether dreaming affects dietary choices, or whether some third factor affects both diet and dreaming. In the case of dream recall, associations with two markers of healthy eating—less evening-eating and higher scores on the *Body-Food Choice Congruence* subscale of the *Intuitive Eating Scale-2—*may reflect adherence to a generally healthy dietary lifestyle. For example, the IES-2 subscale assesses an individuals' choosing of nutritious foods to match their bodies' needs. Thus, items like *Most of the time, I desire to eat nutritious foods; I mostly eat foods that make my body perform efficiently (well);* or *I mostly eat foods that give my body energy and stamina* all suggest active pursuit of a healthy lifestyle that might either induce, or simply include, the regular recall of positive dream content. It is also possible—although unlikely—that individuals who normally recall their dreams frequently are incited by their dreams to make such healthy food choices.

Similarly, the direction of causality among the inter-related measures of nightmares, GI symptoms and evening eating are not resolved by the present findings. GI symptoms and evening eating are clearly factors that might disrupt sleep or bring about dysphoric bodily sensations during sleep that could then find their way into dreams as nightmarish sensations and images. Consistent with this possibility is the finding that a slowly digested (vs. a quickly digested) meal taken late in the evening disrupts sleep via effects on the hypothalamo-pituitary-adrenal axis, and that both types of evening meal increase morning cortisol (Ucar et al., 2021). Such physiological changes could contribute to nightmare production considering that frequent nightmares are characterized by blunted morning cortisol while acute nightmares are associated with elevated cortisol (Hess et al., 2020).

Beyond such alternative effects, it is also feasible that nightmares and dietary symptoms both stem from an as yet unknown third, psychopathological, factor—one not involving anxiety or depression, as these were controlled in most of the present analyses. One possible factor is, alexithymia, a deficit in emotional expression, which is a known correlate of both GI disorders (Porcelli et al., 2003) and dreaming—particularly nightmare severity (Nielsen et al., 2011).

Finally, it should be noted that effects observed in the present study may be due, at least in part, to biases introduced by our exclusive reliance on first-year undergraduate students as participants. These individuals may have had prior academic exposure to topics related to sleep/dreaming, diet, and mental health that influenced how they interpreted or reported the perceived effects of food on sleep and dreaming.

If food choices are indeed a factor causing changes in the nature of dreaming, then interventions focused on diet may well prove useful in treating disturbed dreaming. Our findings suggest that nightmares may turn out to be responsive to diets that minimize the potential for ingesting foods that cause gastric distress, most notably, foods with lactose that cause GI symptoms. Of particular note, there is a growing body of research examining the relationship of diet to post-traumatic stress disorder (PTSD), a major characteristic of which is the frequent occurrence of nightmares. For example, one group (Ke et al., 2023) found that PTSD symptom levels were negatively associated with adherence to a Mediterranean diet, which was in turn linked to the prevalence of certain microbial species in the gut (see also Browne et al., 2021; Rowe et al., 2024). This finding is consistent with emerging evidence of a relationship between the gut microbiome and the central nervous system (the gut-brain axis) which in turn can impact mental health (e.g., MacKay et al., 2024). Another noteworthy example is Irritable Bowel Syndrome, a functional disorder of the gut-brain axis, in which dysregulated serotonergic modulation alters the perception and processing of visceral signals. This dysregulation may both incite GI symptoms and disturb brain circuits involved in emotion regulation (Gros et al., 2021). Since serotonergic dysregulation also alters dream emotions leading, e.g., to nightmares in PTSD (see review in Nicolas and Ruby, 2020), such dysregulation may help explain our findings of a link between diet and nightmares. While most research in this area has examined the relationship of overall dietary patterns to PTSD, the present research suggests that specific foods, such as dairy and sweets, may play a role in exacerbating such symptoms. Dietary approaches to treatment of PTSD might therefore benefit from an assessment of specific food sensitivities.

## 5 Conclusions

The present results further clarify long-standing questions about food's relationship to dreaming and nightmares. In addition to uncovering and controlling for several important confounding factors—gender and psychopathology in particular—we found evidence of food and diet being associated with both dream recall and dream negativity. Specifically, the frequency of dream recall was associated with indicators of healthy eating, i.e., higher scores on a subscale of the *Intuitive Eating Scale-2* and less frequent eating in the evening, while both negative emotional tone of dreams and nightmare severity on the NDI were associated with unhealthy indicators, i.e., combinations of Lactose Intolerance, GI symptoms, less reliance on hunger and satiety cues, and a tendency to eat in the evening. In broad outline, these results are consistent with previous findings that a healthy eating style is associated with more vivid dreaming and higher dream recall while an unhealthy eating style is associated with more disturbing dreams or lower recall (Kroth et al., 2007; Nielsen and Powell, 2015).

Thus, the present results add empirical observations to age-old beliefs that food affects dreaming. They demonstrate that food-dependent dreaming (FDD+) predicts nightmare frequency and severity, with dairy and sweets most commonly being blamed. They show that food sensitivities, Lactose Intolerance in particular, are associated with nightmares–often mediated by GI symptoms. Together, the findings lend support to several hypotheses about food-dependent dreaming that clearly merit further study. Experimental paradigms in which food intake is systematically manipulated could be usefully brought to bear on exploring these hypotheses.

## Data Availability

Ethics rules in our establishments preclude the sharing of data without a formal data-sharing agreement. Requests to access the datasets should be directed to the corresponding author.
