# Supplementary material for: More dreams of the rarebit fiend: food sensitivity and dietary correlates of sleep and dreaming
Source: Front Psychol. 2025 Jul 1;16:1544475. doi: 10.3389/fpsyg.2025.1544475 (PMC12259596; doi:10.3389/fpsyg.2025.1544475)
Supplement: Supplementary file 1 [file Table_1.docx]

**3.1.1. General sleep, dream, dietary and health characteristics (Supplementary Table S1).**

*Supplementary Table S1. Dreaming and nightmare measures for the total sample and women and men separately*

|  | Total | SD-Tot | W | M | SD-W | SD-M | t (gender) | df | *p* |
| --- | --- | --- | --- | --- | --- | --- | --- | --- | --- |
| How often have you recalled your dreams when you awaken?*^ɫ^* | 4.59 | *1.76* | 4.75 | 4.20 | *1.72* | *1.80* | 4.769 | 1056 | *<.001* |
| How often do you recall your nightmares?*^ɫ^* | 3.46 | *1.92* | 3.72 | 2.84 | *1.92* | *1.78* | 7.272^*^ | 700.2 | *<.001* |
| What is the emotional tone of your dreams on average?*^ɫɫ^* | 2.94 | *0.8* | 2.88 | 3.09 | *0.78* | *0.82* | -4.021 | 1055 | *<.001* |
| Nightmare Disorder Index | 2.50 | *3.04* | 2.79 | 1.75 | *3.13* | *2.67* | 5.596^*^ | 763.1 | *<.001* |
| Pittsburgh Sleep Quality Index Item #9*^ɫɫɫ^* | 0.76 | *0.94* | 0.84 | 0.54 | *0.96* | *0.84* | 5.043^*^ | 745.0 | *<.001* |
| *^*^t-test based on unequal variances assumption; ^ɫ^scale: 1: never; 2: <1/mo; 3: about 1/mo; 4: 2-3/mo; 5: about 1/wk; 6: several/wk; 7: almost every morning; ^ɫɫ^scale: 1: very negative; 2: somewhat negative; 3: neutral; 4: somewhat positive; 5: very positive; ^ɫɫɫ^scale: 0: 0/last month; 1: <1/wk; 2: 1-2/wk; 3: 3+/wk* | | | | | | | | | |
