# Supplementary material for: More dreams of the rarebit fiend: food sensitivity and dietary correlates of sleep and dreaming
Source: Front Psychol. 2025 Jul 1;16:1544475. doi: 10.3389/fpsyg.2025.1544475 (PMC12259596; doi:10.3389/fpsyg.2025.1544475)
Supplement: Supplementary file 2 [file Table_2.docx]

**3.1.3. Perceived influence of food on sleep quality (Supplementary Table S2).**

*Supplementary Table S2. Participants naming food groups that produce either better or worse sleep*

|  | Better sleep | |  | Worse sleep | |
| --- | --- | --- | --- | --- | --- |
| Food Group | **N** | **%** | **Food Group** | **N** | **%** |
| Fruit | 166 | *17.6* | **Desserts/Sweets** | 164 | *22.7* |
| Herbal Tea | 126 | *13.4* | **Spicy** | 141 | *19.5* |
| Vegetables | 111 | *11.8* | **Dairy** | 113 | *15.7* |
| Dairy | 96 | *10.2* | **Meat** | 70 | *9.7* |
| Meat | 93 | *9.9* | **Cereals** | 51 | *7.1* |
| Eggs | 72 | *7.7* | **Eggs** | 27 | *3.7* |
| Cereals | 64 | *6.8* | **Pickled** | 26 | *3.6* |
| Nuts | 43 | *4.6* | **Nuts** | 25 | *3.5* |
| Sweets | 32 | *3.4* | **Non-caf. Beverages** | 24 | *3.3* |
| Veg. Protein | 30 | *3.2* | **Fruit** | 23 | *3.2* |
| Spicy | 30 | *3.2* | **Seafood** | 19 | *2.6* |
| Non-caf. Beverages | 29 | *3.1* | **Herbal Tea** | 18 | *2.5* |
| Seafood | 26 | *2.8* | **Vegetables** | 12 | *1.7* |
| Pickled | 23 | *2.4* | **Veg. Protein** | 9 | *1.2* |
| Total | 941 | *100* | **Total** | 722 | *100* |
